# Low PEEP ventilation in TGF-β1 induced lung injury triggers a reversible lung mechanical deterioration without promoting persistent structural damage

**DOI:** 10.1038/s41598-026-61155-9

**Published:** 2026-07-07

**Authors:** Franziska Roeder, Lara-Kristin Steinmetz, Martin Kolb, Ulrich A. Maus, Bradford J. Smith, Lars Knudsen

**Affiliations:** 1 Institute of Functional and Applied Anatomy, Hannover Medical School, Hannover, Germany; 2 Department of Experimental Pneumology, Hannover Medical School, Hannover, Germany; 3 Firestone Institute for Respiratory Health, Department of Medicine, McMaster University, Hamilton, ON Canada; 4 Biomedical Research in Endstage and Obstructive Lung Disease Hannover (BREATH), German Center for Lung Research (DZL), Hannover, Germany; 5 Department of Biomedical Engineering, College of Engineering Design and Computing, University of Colorado Denver | Anschutz, Aurora, CO USA; 6 Department of Pediatric Pulmonary and Sleep Medicine, School of Medicine, University of Colorado Anschutz, Aurora, CO USA

**Keywords:** Acute respiratory distress syndrome, Ventilation induced lung injury, TGF-β1, Recruitment, Atelectasis, Diseases, Medical research, Physiology

## Abstract

TGF-β1-induced lung injury initially results in surfactant dysfunction and alveolar instability (microatelectases). Morphology showed that at end-expiratory pressure of 2cmH_2_O the burden of microatelectasis was high but could be mitigated with a pressure of 8cmH_2_O by 35% without undue strain. Hence, we hypothesized that ventilation of lungs with a higher burden of microatelectasis at positive end-expiratory pressure (PEEP) of 2cmH_2_O triggers more pronounced injury progression compared to PEEP=8cmH_2_O. Mice were randomized to receive either TGF-β1 (AdTGF-β1) for injury induction or empty control vector (AdCl). After a second randomization one week later, mice were ventilated for 4 h with PEEP = 2 or 8cmH_2_O resulting in 4 experimental groups: AdTGF-β1-PEEP2 (*n* = 11), AdTGF-β1-PEEP8 (*n* = 10), AdCl-PEEP2 (*n* = 11) and AdCl-PEEP8 (*n* = 10). During ventilation, every 30 min, Quick-Prime (tissue elastance) and Snap-Shot (dynamic compliance) measurements were performed immediately before and after deep inflations. Finally, lungs were either fixed for stereology (both PEEP2 groups *n* = 6/ both PEEP 8 groups *n* = 5) or broncho-alveolar lavage (*n* = 5) and tissue harvest for transcriptome analyses. During ventilation the increase in tissue elastance was largest in AdTGF-β1-PEEP2 group but reversible by deep inflations. Finally, both AdTGF-β1-groups showed comparable worsening in lung mechanics, and BAL-albumin and neutrophils were elevated. Quantitative morphology showed no differences and transcriptome minimal differences attributable to PEEP-level. Low-PEEP ventilation of AdTGF-β1 lungs with a high burden of microatelectases induced lung mechanical worsening most likely due to progressive alveolar derecruitment. This injury pattern was reversible upon deep inflations and not linked with signs of persistent lung injury such as vascular leakage, edema or inflammation.

**Supplementary Information:**

The online version contains supplementary material available at 10.1038/s41598-026-61155-9.

## Introduction

Mechanical ventilation of pre-injured lungs presents a significant clinical challenge due to the altered micromechanical properties of the pulmonary parenchyma. A major concern in this context is the risk of ventilation-induced lung injury (VILI), which can aggravate pre-existing pulmonary damage through increased stresses, and strains, and energy dissipation in the lung. The mortality rate among patients with acute respiratory distress syndrome (ARDS), even after implementation of protective ventilation strategies remains exceedingly high, ranging approximately between 30 and 40%^1^. Both morbidity and mortality in mechanically ventilated patients are influenced by ventilator parameters such as positive end-expiratory pressure (PEEP), tidal volume and the use of recruitment or deep inflation maneuvers. Consequently, considerable efforts have been directed toward personalizing mechanical ventilation by adjusting these parameters to the current mechanical conditions of the lung, particularly pulmonary compliance^2,3^.

In healthy lungs, alveoli are stabilized by the elastic fibers and the surfactant system, so that they remain patent throughout the respiratory cycle^4^. In pre-injured lungs, inflammation and interstitial- or intra-alveolar edema are often accompanied by surfactant dysfunction. This dysfunction increases alveolar surface tension, thereby promoting alveolar collapse. Within the heterogeneous pathology characteristic of acute lung injury, three distinct alveolar phenotypes have been identified^5^. First, healthy alveoli remain open throughout the respiratory cycle. Besides healthy areas there are regions in which alveoli are subject to repetitive recruitment and derecruitment during each breath and regions where alveoli remain persistently derecruited or filled with edema, rendering them non-recruitable. These collapsed or non-recruitable alveoli form microatelectases, by folding the interalveolar septa onto each other. Groups of derecruited alveoli (microatelectases) affect the neighboring alveoli through alveolar interdependence meaning that these neighboring alveoli are at risk for local volutrauma^4^. The microatelectases act as stress concentrators, as the radial tensile forces originating from these regions impose mechanical stress on the neighboring alveoli^6^. Consequently, the interalveolar septa bordering these alveoli become overstretched, as they must accommodate an increased volume displacement caused by the collapsed alveoli. This causes a volutrauma, which can release pro-inflammatory cytokines leading to a biotrauma^7^.

Because of these challenges, lungs suffering from ARDS are ventilated using lung-protective strategies to preserve function and prevent additional damage. Based on the “baby lung concept” this includes using a low tidal volume of approximately 6 ml/kg body weight to avoid volutrauma^8–10^. Evidence regarding the protective PEEP is still inconclusive, with some studies advocating higher pressures to recruit atelectatic areas^11^, while others recommend lower pressures to avoid alveolar overdistension^12^. In other words, the PEEP adjustment is a compromise between recruitment of distal airspaces and static strain of functional lung parenchyma. The use of electrical impedance tomography or computed tomography to determine an optimal PEEP during ventilation balancing recruitment against static strain has also been investigated in recent studies demonstrating the feasibility of this approach^13–15^. The adjustment of PEEP based on these techniques may reduce VILI and improve respiratory mechanics.

The aim of our study was to investigate the effects of two different PEEP levels during mechanical ventilation on healthy and pre-injured mouse lungs. Lung injury was induced by an adenoviral TGF-β1 vector, which causes acute lung injury within seven days. This acute lung injury is characterized by surfactant dysfunction, the occurrence of microatelectasis, and mild alveolar edema. We selected PEEP levels based on our previous work^16^. A PEEP of 2 cmH₂O represented the injurious setting, which according to our prior study was linked with a high burden of collapsed alveoli and thus potential stress concentrators that might raise the risk for VILI. In contrast, a PEEP of 8 cmH₂O represented in this study the protective setting: it effectively reduced the burden of microatelectases without undue overdistension of the acinar airspaces. In other words, stress concentrators were substantially reduced with PEEP = 8 but not 2 cmH_2_O. Hence, we hypothesized that mechanical ventilation with PEEP = 2 cmH_2_O aggravates lung injury on a larger scale than mechanical ventilation with PEEP = 8 cmH_2_O.

## Methods

### Animal model

This study used 10 to 11 week old female C57BL/6J mice (Janvier, Le Genest-Saint-Isle, France). The body weight ranged from 17.3 g to 24 g with a mean of 20.4 g and a standard deviation (SD) of 1.2 g. To reduce variability in the stereological data, only female mice were used in this study. All procedures were performed in accordance with the Ordinance for Animal Experiments and approved by the LAVES (Lower Saxony State Office for Consumer Protection and Food Safety, approval number 21/3829). The mice (*N* = 42) were randomly divided into two groups: one group served as the control (*N* = 21), while the other group of mice was exposed to adenoviral vector mediated TGF-β1 gene transfer (*N* = 21). Planning, execution, and analysis of experiments were carried out in accordance with the ARRIVE guidelines^17^. For the intratracheally instillation, the mice were anesthetized by intraperitoneal (i.p.) anesthesia consisting of Xylazine/Ketamine, (Rompun 2%, Bayer, Leverkusen, Germany and Ketamin, CP-Pharma, Burgdorf, Germany) and orotracheally intubated. Afterwards, 10^8^ plaque-forming units of an adenoviral vector carrying the TGF-β1 gene (AdTGF-β1) diluted in 50 µl PBS, were administered via the tracheal cannula^16^. The randomized control group was treated with 50 µl PBS (phosphate-buffered saline) containing empty adenoviral control vector (AdCl). The animals were observed and weighed daily. The mean loss of body weight within 7 days was 1.74 g (± 1.35 g) in AdTGF-β1 and 0.1 g (± 0.53 g) in AdCl. None of the mice lost more than 20% of baseline body weight. One week after the instillation, the mice were ventilated for four hours, based on randomization, either with PEEP = 2 cmH_2_O or PEEP = 8 cmH_2_O. The randomization was achieved by selecting the mice from the cage by rolling a cube. If the cube showed an even number, all mice labelled with an even number were ventilated with PEEP = 2 cmH_2_O and others with PEEP = 8 cmH_2_O. It was the other way round if the cube showed an uneven number. Together with the randomized treatment groups, there were in total four groups: AdTGF-β1-PEEP 2 (*n* = 11), AdTGF-β1-PEEP 8 (*n* = 10), AdCl-PEEP 2 (*n* = 11) and AdCl-PEEP 8 (*n* = 10). After the ventilation the lungs were alternating used either for lung stereology (both PEEP2 groups *n* = 6/ both PEEP 8 groups *n* = 5) or for analyses of the BAL and lung parenchyma (each group *n* = 5). If an animal died during ventilation, it was excluded from the subsequent analysis. For stereological investigations the vascular perfusion fixation at a defined airway opening pressure is critical. Lungs were excluded from further stereological investigation based on the following criteria: (1) at light microscopic level the alveolar capillary network was collapsed, indicating inappropriate vascular perfusion fixation. (2) The lung volume measured by fluid displacement was equal or lower than the volume of air that was used to inflate the lung at an airway opening pressure of 5 cmH2O on expiration as measured by FlexiVent. Volume loss would have critically affected the stereological data as very important study endpoints and occurs due to air leakage that might happen during preparation, e.g. due to incidentally cutting into the lung or insufficient ligation of the trachea.

### Lung mechanics measurements

**Fig. 1:**
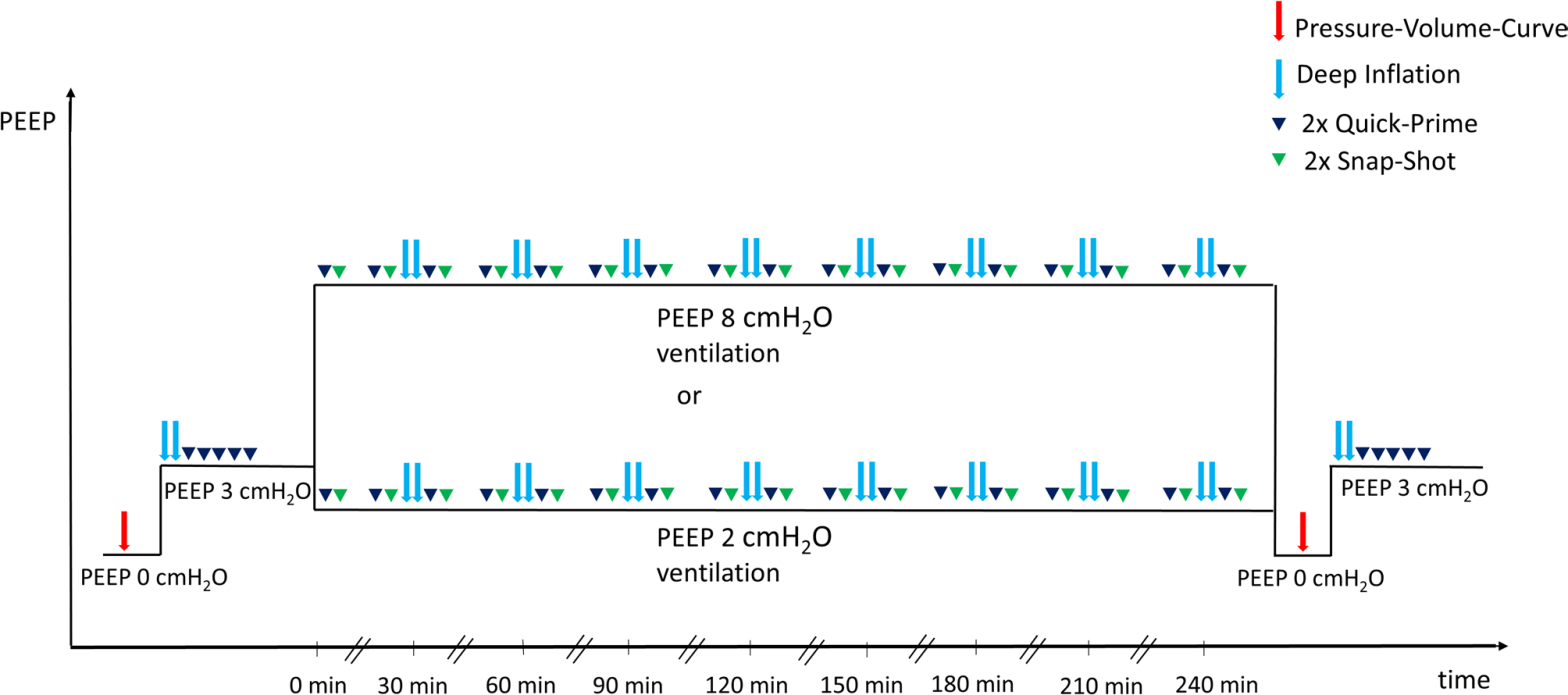
FlexiVent script. The script was executed using AdTGF-β1 and AdCl mice, that were randomized for the main block to be ventilated either with PEEP = 2 or 8 cmH_2_0. The protocol was identical at the beginning and end for all groups. During PEEP 2 cmH_2_O or PEEP 8 cmH_2_O ventilation measurements were performed every 30 min. At the beginning and at the end of the 4 h period of either PEEP 2 or 8 cmH_2_O, pressure-controlled, quasi-static pressure volume loops were recorded during a brief period of PEEP = 0 cmH_2_O ventilation. After that, derecruitability tests consisting of 2 deep inflations and repetitive Quick-Prime perturbations were carried out during PEEP = 3 cmH_2_O ventilation in all study groups.

For the ventilation, both the control and the AdTGF-β1 mice, were anesthetized intraperitoneally (i.p.) with fentanyl (Piramal, Hallbergmoos, Germany)) (75 µg/kg body weight), midazolam (Ratiopharm, Ulm, Germany) (2 mg/kg body weight), and medetomidine (cp-Pharma, Burgdorf, Germany) (750 µg/kg body weight)^18^. For the subsequent procedure, the animals were again randomized into two groups and ventilated with different PEEP levels. Half of the mice were ventilated with a PEEP of 2 cmH_2_O, while the other half received ventilation with a PEEP of 8 cmH_2_O. Following anesthesia, a tracheotomy was performed and the mice were connected to the FlexiVent ventilator (FX1 mouse module, SQIREC, Montreal, Canada) that is controlled by the flexiWare software (flexiWare Version 8.2, SQIREC, Monreal, Canada). The script of the ventilation protocol and the time of the measurements is visualized in Fig. 1. The mice were closely monitored for clinical signs of impending death during mechanical ventilation such as loss of palpable heart beats and development of a pale skin or tongue. In support of these clinical signs, death was usually inferred from the ongoing lung mechanical measurements, particularly through considerable and non-reversible increase in peak inspiratory pressure (Pmax), which indicated decreasing compliance of the respiratory system after heart arrest and breakdown of the circulation.

To ensure comparability across all experimental groups, measurements obtained at the beginning and end of the protocol were performed at a PEEP level of 3 cmH_2_O. The respiratory rate was 150/min, the inspiratory to expiratory ratio 1:2 and the tidal volume 10 ml/kg body weight. The body weight used for mechanical ventilation corresponded to the weight recorded prior to tracheal instillation of the adenoviral vectors at baseline. Each of these initial and final measurement blocks began with quasi-static pressure-controlled pressure–volume loops. Recording started at an onset pressure of 0 cmH_2_O and thus during zero-PEEP ventilation. The aim of this approach was to collect data over a maximal range of lung volumes above residual volume. Subsequently, derecruitability tests were performed during PEEP = 3 cmH_2_O ventilation. Two deep inflations were administered followed by ten Quick-Prime perturbations at an onset pressure of 3 cmH_2_O with a periodicity of 30 s. During each deep inflation, airway pressure was increased to 30 cmH_2_O over 3 s and held at this level for an additional 3 s. The Quick-Prime perturbation, based on the forced oscillation technique (FOT), records impedance spectra to which the constant-phase-model is fit to calculate tissue elastance (H), tissue damping (G) and Newtonian resistance (Rn). As the outputs of FOT fitted to the constant phase model are highly dependent on the PEEP level, derecruitability tests were performed at PEEP = 3 cmH_2_O for all groups, enabling direct comparison between groups ventilated with PEEP = 2 or 8 cmH_2_O throughout the main ventilation study. The use of two deep inflations prior to repetitive FOT perturbation has previously been shown to effectively normalize volume history^19,20^. Measurements with a coefficient of determination (COD) of 0.8 and above, indicating an adequate model fit, were accepted for further analysis while those with a COD below 0.8 were excluded from further investigations. After completion of the first block, the subsequent measurements were carried out at either PEEP = 2 cmH₂O or PEEP = 8 cmH₂O, depending on the group to which the animals had been randomized. During long-term ventilation respiratory rate was 190 /min, the inspiratory-to-expiratory ratio was 1:2 and the tidal volume was 8 ml/kg. Each ventilation block started with two Quick-Prime measurements with an onset pressure of either 2 or 8 cmH2O, depending on the ventilation protocol, to which the mice were randomized. The Quick-Prime measurements were followed by two Snap-Shot maneuvers, in which a single-compartment model was applied to determine the dynamic compliance (C_dyn_) and its inverse, the dynamic elastance. The Snap-Shot perturbation, depending on the group assignment, also starts with an onset pressure of 2 or 8 cmH_2_0 and records 1 dynamic volume controlled sinusoidal pressure-volume loops with a respiratory rate of 60/min and a tidal volume of 10 ml/kg body weight. After 30 min of ventilation, both sets of measurements were repeated, followed by two deep inflations to recruit the lung parenchyma. Following recruitment, Quick-Prime and Snap-Shot measurements were repeated to allow comparison of parameters before and after lung recruitment. This sequence of measurements, including the deep inflations, was repeated every 30 min during the 4-hour long-term ventilation period. At the conclusion of the experiment, the initial measurement block at PEEP 3 cmH₂O was repeated to assess potential changes over time which was calculated as the difference in H measured after and before the 4 h period of PEEP = 2 or 8 cmH_2_O ventilation.

### Fixation, sampling and tissue processing

After completion of the experimental protocol, the mice received an intraperitoneal injection of fentanyl (Piramal, Hallbergmoos, Germany, 225 µg/kg body weight) and midazolam (Ratiopharm, Ulm, Germany, 6 mg/kg body weight) to deepen narcosis and to perform a laparotomy. Afterwards the abdominal aorta was dissected and incised so that the animals died from exsanguination. Then the lungs were fixed by vascular perfusion at end-expiration by maintaining an airway opening pressure of 5 cmH₂O allowing studying of the lung at a lung volume within the range in which breathing takes place based on a recent recommendation^21^. The pulmonary circulation was flushed via the right ventricle with 0.9% NaCl–heparin solution (25,000 IU/mL; Ratiopharm, Ulm, Germany) at a hydrostatic pressure of 30 cmH₂O while the left atrium was opened. After rinsing the lungs, perfusion fixation was performed through the right ventricle using a fixative mixture containing 1.5% paraformaldehyde and 1.5% glutaraldehyde in 0.15 M HEPES buffer. The lungs were dissected and stored overnight in the same fixative solution. For the following analysis, the investigators were blinded and did not know which group each sample belonged to.

For stereological analysis, lung volume was determined using Archimedes’ principle of water displacement^22^. Subsequent processing up to the preparation of stained sections was performed as previously described^22,23^. Accordingly, a systematic uniform random sampling method was applied, and the lungs were cut from the apex to the basis in 1 mm thick, transversal lung slices. These were numbered and based on random number table either every even or uneven numbered slices were embedded in Technovit 8100 (Kulzer Heraeus, Wehrheim, Germany) prior to cutting into 1.5 μm thick histological sections. For stereological analysis, the first and fourth of a consecutive series of sections were mounted on a glass slide, stained with Toluidine Blue and subsequently cover-slipped. The distance of 4.5 μm from the top of the first section to the top of the fourth section is optimal for using the stereological physical disector method to accurately determine the number of open alveoli. The slides were digitalized with the AxioScan (Zeiss, Oberkochen, Germany) at 20 x primary magnification so that the stereological analyses could be done with the newCAST software (Version 3.2, Visiopharm, Hoersholm, Denmark). Digitalized slides were imported into the newCAST software, and 100 to 200 fields of view were generated based on a systematic uniform area sampling algorithm. The exact stereological procedure was performed as described in detail by Roeder et al.^16^. In brief, the randomized fields of view were subjected to point and intersection counting to determine volume fractions and surface densities of structures of interest within a defined reference volume. At the first step, the reference volume equaled the lung volume V(lung) and the volume fraction of lung parenchyma Vv(par, lung) within the lung volume was determined. By multiplication of the volume fraction and the reference space the absolute volume of lung parenchyma per lung was obtained (V(lung) x Vv(par, lung) = V(par, lung)). In the next step, the lung parenchyma V(par, lung) became the reference space for point and intersection counting. This way, the volume of alveolar airspaces (Vv(alv, par) x V(par, lung) = V(alv, par)), ductal airspaces (Vv(duct, par) x V(par, lung) = V(duct, par)) and interalveolar septa (Vv(sep, par) x V(par, lung) = V(sep, par)) were determined. Using in the third step the volume of interalveolar septa as reference space, the volume fractions of recruited septa and derecruited septa (= collapsed septa) were determined as outlined in detail in Roeder et al. 2024^16^. In addition, intersection counting was used to determine the surface area density of alveoli within lung parenchyma. The arithmetic mean thickness of interalveolar septa (τ(sep)) was calculated as a volume-to-surface ratio (τ(sep) = 2 x V(recsep, par)/S(alv, par)). Finally, the number of open alveoli (N(alv, par)) was quantified using the physical disector method^16^.

### Transmission electron microscopy

For electron microscopy, tissue was sampled based on a systematic uniform random sampling design to obtain at least six representative tissue blocks per lung^22^. These blocks had an edge length of approximately 1 mm and were processed and embedded in epoxy resin (Epon^®^, Polyscience, Hirschbergan der Bergstr., Germany) based on established protocols^23^. Embedded samples were sectioned in ultrathin sections of a thickness of 60 nm (Ultracut S, Leica Reichert, Wetzlar, Germany). These samples were qualitatively investigated by transmission electron microscopy (Morgagni, FEI, Eindhoven, The Netherlands) with a special focus on interstitial and alveolar edema, integrity of the blood-gas barrier and the ultrastructure of the intra-alveolar (lamellar bodies in alveolar epithelial type 2 cells) and intracellular surfactant system.

### Biochemical analysis

For biochemical analyses, mice were disconnected from the FlexiVent and the vasculature of the lungs was rinsed with 0.9% saline. The BAL was taken by flushing the lung three times with 1 ml 0.9% NaCl. BAL fluids were used for determination of total albumin concentrations using commercially available kit according to the manufacturer´s instructions (Mouse Albumin ELISA Kit E99-134 Bethyl, Montgomery, USA). The BAL samples of four unventilated mice from each group (AdCl and AdTGF-β1) from our prior study^16^ were reanalyzed together with those from the ventilated mice of the present study to investigate if the different mechanical ventilation protocols aggravate vascular leakage and thus increase in albumin levels in BAL. Cytospots were also prepared from the BAL fluid samples. For this purpose, the BAL fluid was centrifuged at 1500 RPM (revolutions per minute) and 4 °C for 10 min. Subsequently, the cell suspension was stained with trypan blue dye to assess viability, and total cell counts were performed. A total of 50,000 cells were then cytocentrifuged onto microscope slides and stained using Diff-Quick stains. The prepared slides were scanned with the Axioscan system, and cellular constituents were analyzed. Lung tissue was cut into small pieces and was frozen in liquid nitrogen and stored for the transcriptome analysis.

### Transcriptome analysis

Transcriptome analysis was performed on ventilated mice, comparing the PEEP 8 cmH₂O group to the PEEP 2 cmH₂O group within each treatment condition. In addition, transcriptome profiling was conducted in non-ventilated mice that had undergone the same pretreatment regimen. These non-ventilated animals were part of our previous study, focusing on structure-function relationships in non-ventilated AdCl and AdTGF-β1 mice, those transcriptome data have not yet been published (Roeder et al. 2024). To enable comprehensive insights, the transcriptomic profiles of ventilated mice were further compared to those of non-ventilated mice from the same treatment group. This cross-comparative approach allowed for investigation of the effects of the different adenoviral vectors and the different mechanical ventilation protocols on transcriptome profiles.

For the transcriptome analysis, the frozen tissue was thawed overnight in RNAlater ICE (Thermo Fischer, Darmstadt, Germany), and RNA was extracted using the NucleoSpin RNA kit (Macherey & Nagel, Düren, Germany) following the manufacturer’s instructions. The RNA concentration was measured with the Nanodrop (Thermo Fischer, Darmstadt, Germany) and the following bulk transcriptome processing was performed by the genomics core facility of Hannover Medical School.

### Library generation, quality control and quantification

For each sample, 500 ng of total RNA was used as input for ribosomal RNA depletion utilizing the NEBNext rRNA Depletion Kit (Human/Mouse/Rat, 96 reactions; E6310X; New England Biolabs). This was followed by the generation of stranded cDNA libraries employing the NEBNext Ultra II Directional RNA Library Prep Kit for Illumina (E7760L; New England Biolabs). All procedures were carried out according to the manufacturer’s protocol (User Manual E7760, v1.0_02-2017; NEB), with the exception that reaction volumes were scaled down to two-thirds of the recommended amounts.

cDNA libraries were uniquely barcoded using a dual-indexing strategy with NEBNext Multiplex Oligos for Illumina – 96 Unique Dual Index Primer Pairs (6440 S; New England Biolabs). Libraries were amplified via eight cycles of final PCR. At the end of the protocol, an additional purification step was incorporated using 1.2× Agencourt AMPure XP Beads (A63881; Beckman Coulter, Inc.).

For the assessment of the distribution of the fragment sizes of each library the Bioanalyzer High Sensitivity DNA Assay (5067 − 4626; Agilent Technologies) was used, and the library quantification was performed with the Qubit dsDNA HS Assay Kit (Q32854; Thermo Fisher Scientific).

### Library denaturation and sequencing run

Each uniquely barcoded library was adjusted to an equal molar concentration before pooling, so that each library representing approximately 5.6% of the total flow cell capacity. Following the Illumina Denature and Dilute Libraries Guide (Document No. 15048776 v02), the denaturation of the library pool lead to a concentration of 1.8 pM. A volume of 1.3 mL of the denatured pool was loaded onto an Illumina NextSeq 500 High Output Kit v2.5 flow cell (75 cycles, 400 million clusters; catalog number 20024906). The Sequencing started with the parameters: Sequence reads of 38 bases each (Read 1 and Read 2) and dual index reads of 8 bases each (Index 1 and Index 2).

### Data processing

With bcl2fastq Conversion Software version v.2.20.0.422 (Ilumina Solution Center, Berlin, Germany) the BCL files could be converted to FastQ files. The data processing was performed by use of nfcore/rnaseq(v.3.9). Reference data were taken from GENCODE.org (Mus musculus: GRCm39; release M34). Differential expression and normalization analyses were conducted on the RCUGenomics internal Galaxy server (v20.05) at Hannover Medical School, Germany, using DESeq2 (Galaxy Tool v2.11.40.6, https://toolshed.g2.bx.psu.edu/view/iuc/deseq2/0696db066a5b). Default parameters were applied, except that *“Output normalized counts table”* was set to *Yes*, and all additional filters were disabled (*“Turn off outliers’ replacement*,*” “Turn off outliers filtering*,*”* and *“Turn off independent filtering”*).

### Statistical analysis

For sample size estimation, a power analysis using G*power was applied^24^. The primary endpoint was tissue elastance at the end of the study, as this parameter has been shown to correlate with injury progression and stereological parameters relevant to quantifying acute lung injury including VILI. Estimation was based on a F test ANOVA design considering two factors (AdCl vs. AdTGF-β1 and PEEP 2 vs. PEEP8 cmH_2_O) and four groups. Taking a statistical power of 0.8 and a significance level α = 0.05 (error probability) as a basis, a total sample size of 45 animals was estimated^6,25,26^. The statistical analysis of data was performed with GraphPad Prism (Version 10, Graphpad Software, Inc., Bosten, USA). After testing for normal distribution, the two-way ANOVA was used to investigate significant effects on the data of two factors, such as. “treatment” (AdCl vs. TGF-β1) and “pressure” (PEEP 2 and 8 cmH_2_O) for independent measurements or “time” and “pressure” for dependent measurements of lung mechanical data over time. If significant effects of either of these factors were identified, a post-hoc Tukey test was added. The figures were prepared with GraphPad Prism (Version 10, Graphpad Software, Inc., Bosten, USA) and the software Qlucore Omics Explorer (Version 3.11, QLUCORE, Lund, Sweden). The levels of statistical significance are *p < 0.05, **p < 0.01, ***p < 0.001 and ****p < 0.0001.

## Results

### Survival

Several mice died during the four-hour ventilation. The mortality rates were one out of 11 and 2 out of 12 for AdCl PEEP 8 and AdCl PEEP 2 group, respectively. In the AdTGF-β1 PEEP 8 group 4 mice died out of 14 and in the AdTGF-β1 PEEP2 group 6 out of 16 died during mechanical ventilation. Despite tendencies, there were no significant differences (*p* = 0.23, Mantel-Cox) between the four study groups. Main reason for death was respiratory failure followed by cardiac arrest.

### Lung mechanics data

**Fig. 2:**
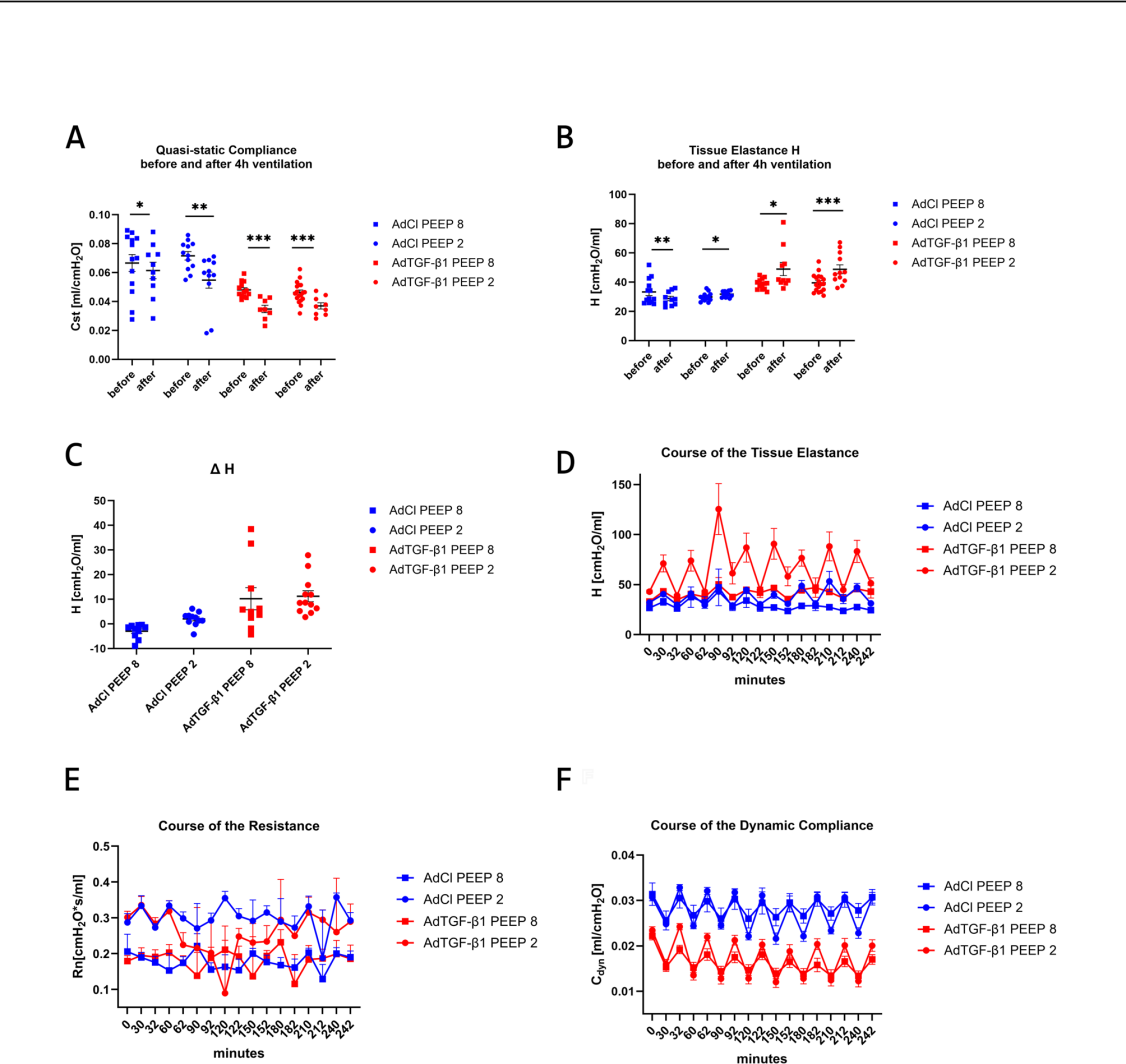
Lung mechanics. The quasi-static compliance (Cst) decreased during the four hours of ventilation in all study groups (**A**). Tissue elastance measured at PEEP = 3 cmH_2_O before and after 4 h of mechanical ventilation (H) showed increases in both AdTGF-β1 groups and AdCl PEEP2 but a decrease in AdCl PEEP8 (**B**). For each subject, the difference in H measured before and after four-hour ventilation is not influenced by the PEEP level (**C**). The courses of H (**D**), Newtonian resistance Rn (**E**) and dynamic compliance C_dyn_ (**F**) determined during PEEP = 2 or PEEP = 8 cmH_2_O ventilation are visualized as function of time. H increased over time and could be reduced by deep inflations given every 30 min, an observation that was most pronounced in the AdTGF-β1 PEEP 2 group in contrast to the other groups. Rn did not show reproducible effects upon deep inflation but was in general larger in PEEP = 2 cmH_2_O compared to PEEP = 8 cmH_2_O ventilated lungs. Reduced C_dyn_ could be increased by deep inflations and demonstrated in general larger values in AdTGF-β1 than in AdCl. For A and B, a two-way ANOVA was performed to investigate the effects of time (before and after) and PEEP level. In C a two-way ANOVA was performed taking the factors pretreatment (AdCl vs. AdTGF-β1) and the PEEP-level into consideration. In mean and standard error of the mean of 10–11 independent measurements are shown. Statistically significant differences were indicated as follows: *p < 0.05, **p < 0.01, ***p < 0.001.

The quasi-static compliance (Cst) showed a significant decrease after four hours of ventilation within each subgroup at both PEEPs (Fig. 2A). The Cst after ventilation was not affected by PEEP neither in the AdCl nor AdTGF-β1 groups. In AdCl PEEP 8 a ventilation-induced decrease in H was observed while a significant increase in H was seen in all other subgroups (Fig. 2B and C). However, as with Cst, the PEEP level did not influence the final tissue elastance in AdCl or AdTGF-β1.

The time course of tissue elastance H during ventilation with PEEP = 2 or 8 cmH_2_O exhibited a zigzag-like pattern due to deep inflations that were applied every half hour. Within the time interval between two deep inflations H increased and was reduced by the next deep inflation to near baseline levels. Compared to AdTGF-β1 PEEP 2, where this gain in H was very steep, the healthy subgroups (AdCl PEEP 2 and PEEP 8) as well as the AdTGF-β1 PEEP 8 subgroup showed less severe increases in H between 2 deep inflations. The deep inflations, however, effectively reduced H almost to the initial values independent from the group assignment. Comparing the final tissue elastance H data at the end of 4 h of mechanical ventilation before and after deep inflations showed PEEP (*p* < 0.001) and pre-treatment effects (*p* < 0.05). Significantly larger reductions in H were induced in AdTGF-β1 PEEP 2 than in AdTGF-β1 PEEP 8 (34.92 ± 25.70 vs. 6.61 ± 2.16 cmH_2_O/ml, *p* = 0.0017). The reductions in H due to final deep inflations in the AdCL group on the other hand was not significantly affected by PEEP level (12.73 ± 3.11 vs. 3.13 ± 2.24 cmH_2_O/ml *p* = 0.2672). The Newtonian Resistance (Rn) was also determined by fitting the constant phase model to the impedance spectra from the FOT perturbations. This data reflects the resistance of the conducting airways and are given in Fig. 2E. Rn is not affected by the pre-treatment of the lungs with different adenoviral vectors. Instead, both AdCL and TGF-β1 ventilated with PEEP = 8 cmH2O tended to have smaller Rn values than ventilated with PEEP = 2 cmH_2_O across long phases of the mechanical ventilation period. The dynamic compliance C_dyn_ was calculated, based on the single compartment model, from dynamic, volume-controlled sinusoidal pressure-volume loops and data is shown in Fig. 2F. Independent from PEEP level 2 or 8 cmH_2_O, AdCl groups had larger C_dyn_ values compared to AdTGF-β1. In general, higher fluctuations in C_dyn_ could be observed with PEEP = 2 cmH2O in both AdCl and AdTGF-β1 due to deep inflations. While before a deep inflation C_dyn_ tended to be smaller in PEEP 2 groups than in PEEP 8 groups, it was the other way round after the deep inflation: C_dyn_ tended to be larger during PEEP = 2 cmH_2_O compared to PEEP = 8 cmH_2_O ventilation.

**Fig. 3:**
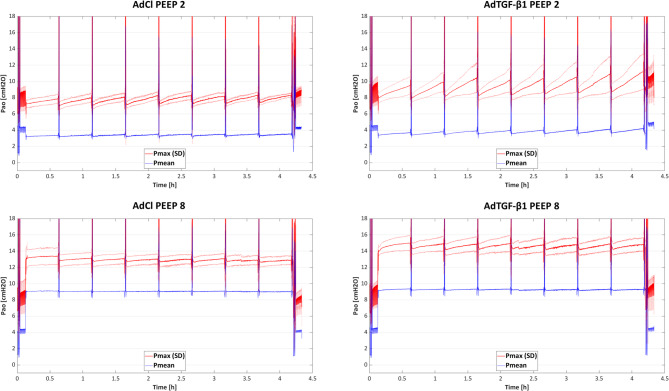
Data streams showing mean airway opening pressure (blue, Pmean) and maximal airway opening pressure (red, Pmax) with standard deviation (SD) as a function of time. The Y-axis shows the airway opening pressure (Pao). The increase in Pmax between two deep inflations was more pronounced in the AdTGF-β1 groups compared to controls, especially in the PEEP 2 group, which exhibited the largest increase. Before final derecruitability tests, Pmax was significantly larger in PEEP 2 groups compared to PEEP 8 groups in both AdCL (*p* = 0.001) and AdTGF-β1 (*p* = 0.028). A two-way ANOVA was performed taking the factors pretreatment (AdCl vs. AdTGF-β1) and the PEEP-level into consideration.

Figure 3 shows the peak inspiratory (Pmax) and mean airway opening pressures over the course of the experiment. Between two deep inflations Pmax increased in all study groups with different gradients and, like tissue elastance, was reduced by a deep inflation. The most rapid increases in Pmax were documented for AdTGF-β1 PEEP 2. Moreover, the slope became steeper from interval to interval, so that the steepness was most pronounced in the final half-hour of PEEP = 2 cmH_2_O ventilation in AdTGF-β1. The driving pressure is the difference between end-inspiratory airway opening pressure (usually determined during an inspiratory hold at the bedside) and PEEP. The difference between peak inspiratory pressure (Pmax) and PEEP (ΔP) is not identical to the driving pressure used in clinical practice, but it may be considered an approximation.

In the AdTGF-β1 PEEP 2 group, the mean ΔP increased during the 30-minute intervals between deep inflations. During the first 30 min of ventilation at PEEP = 2 cmH_2_O, the mean ΔP rose from 6.0 (± 1.5) to 7.4 (± 1.2) cmH_2_O, whereas the increase was much steeper during the final 30-minute interval, rising from 6.7 (± 1.0) to 9.2 (± 2.1) cmH_2_O. In the other study groups, the increase in mean ΔP was less steep and generally more stable over time. In AdTGF-β1 PEEP 8, ΔP was between 6 and 7 cmH_2_O during both the initial and final 30-minute intervals between deep inflations. At the end of the ventilation protocol and before deep inflation, ΔP was significantly higher in AdTGF-β1 PEEP 2 than in AdTGF-β1 PEEP 8 (9.2 ± 2.1 cmH_2_O vs. 6.2 ± 0.7 cmH_2_O, *p* = 0.0281).

In general, ΔP was much more stable in AdCL groups. In AdCL PEEP 2 ΔP increased during the first 30 min interval from 5.2 (± 0.5) to 5.9 (± 0.4) cmH_2_O and during the last 30 min interval from 5.4 (± 0.3) to 6.3 (± 1.0) cmH_2_O. Most constant were ΔP values in AdCL PEEP 8 where slight increases from 5.1 (± 1.1) to 5.4 (± 1.0) cmH_2_O in the initial and from 4.6 (± 0.6) to 4.8 (± 0.6) cmH_2_O in the final 30-minutes interval between deep inflations could be observed. Although the differences in ΔP at the end of the ventilation period were small between AdCL PEEP 2 and AdCL PEEP 8 they were statistically significant (6.3 ± 0.3 cmH_2_O vs. 4.8 ± 0.6 cmH_2_O, *p* = 0.001).

### Morphology

**Fig. 4:**
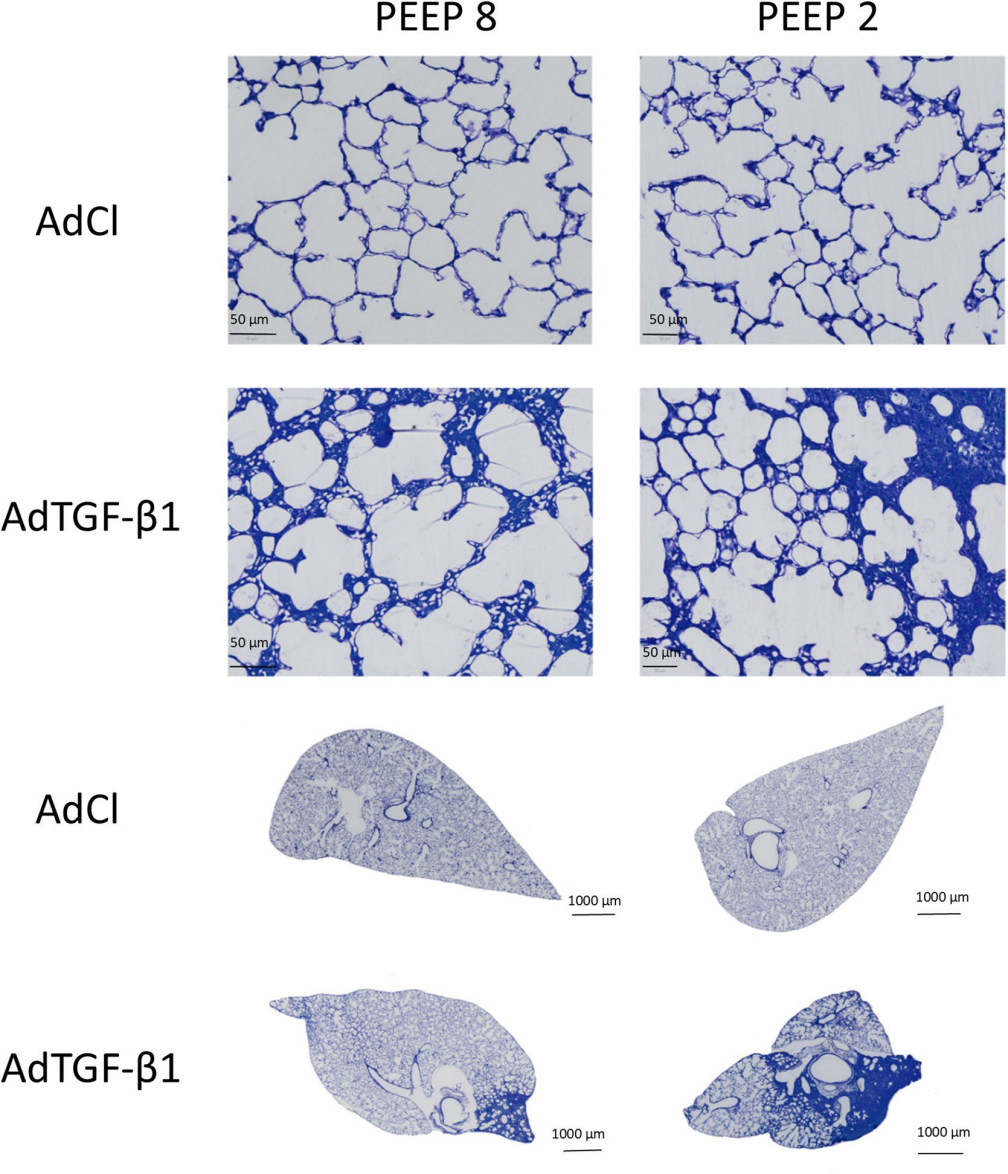
Lung morphology. Light microscopic sections at both overview and higher magnification reveal differences between the two treatment groups in the extent of collapsed regions, where alveoli are collapsed and septa are folded, appearing as dark blue areas. No differences were observed between different PEEP levels.

The light microscopic sections (Fig. 4) clearly revealed structural differences between the two treatment groups, AdCl and AdTGF-β1. The AdTGF-β1 group displayed more collapsed alveoli and microatelectases, visible as dark blue areas in the overview images (bottom rows) and in the higher magnification panels (top rows). The overview indicated that these collapsed regions are distributed heterogeneously rather than homogeneously over the sections. The corresponding images with higher magnification showed regions where the septa are folded on each other into multiple layers of tissue because of alveolar collapse. Similar heterogeneous areas of collapsed alveoli were present in both AdTGF-β1 PEEP groups. In contrast, the alveoli of the AdCl groups appeared more stable, with collapsed regions occurring much less frequently. The images of the AdCl mice in the top row showed septa containing well-preserved capillaries, maintained by perfusion fixation. There were also no observable differences between the different PEEP levels.

### Stereology

**Fig. 5:**
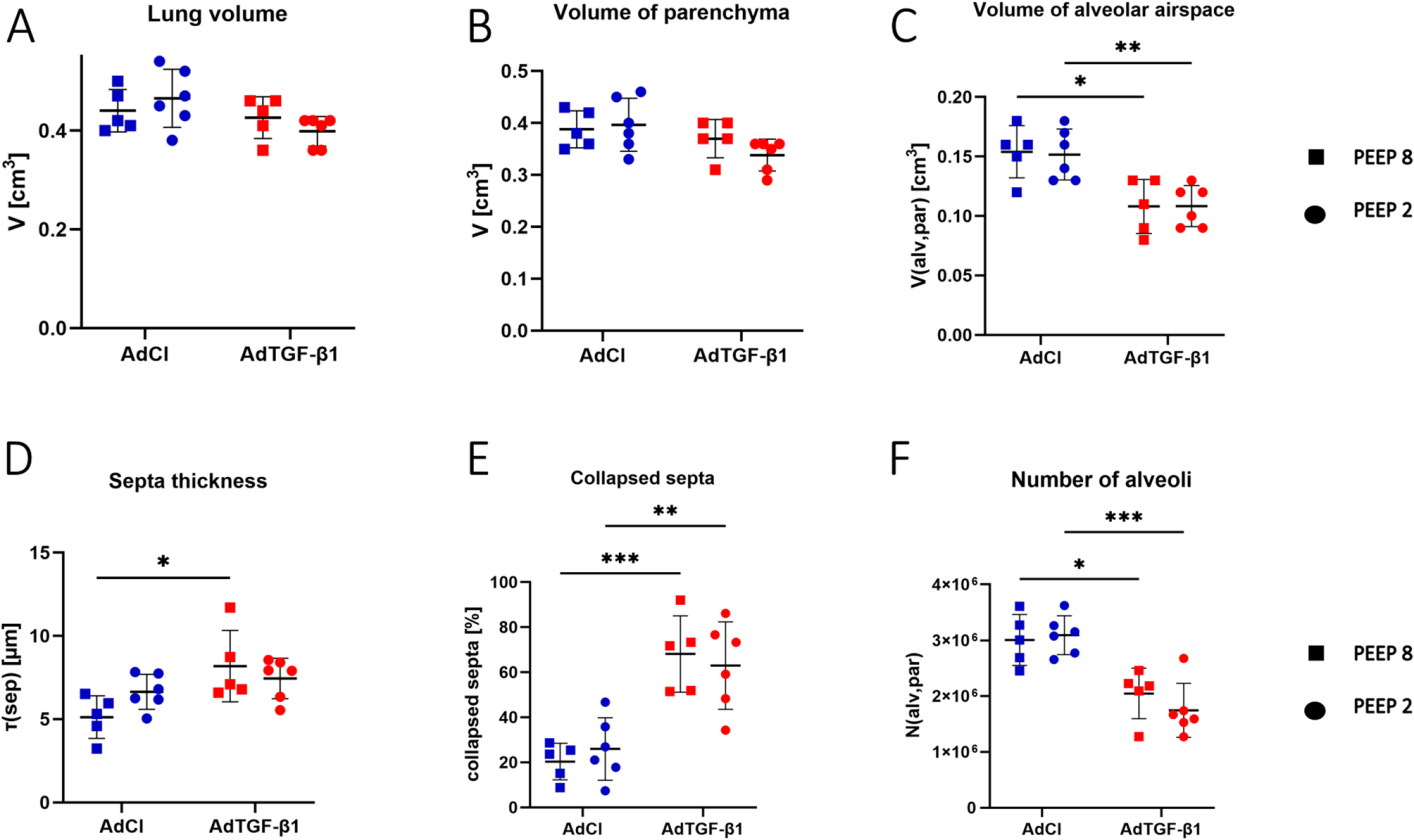
Lung stereology of mice ventilated for four hours. Lung volume (**A**) and parenchymal volume (**B**) did not differ between treatment groups or PEEP levels. In contrast, alveolar airspace volume (**C**), septal thickness (**D**), the percentage of collapsed septa (**E**) and the number of open alveoli (**F**) showed significant differences between treatments, but no significant differences were observed between PEEP levels within the same treatment group. Individual measurements, mean and standard deviation are illustrated. For statistics, a two-way ANOVA was performed. Statistically significant differences were indicated as follows: *p < 0.05, **p < 0.01, ***p < 0.001.

The stereological analysis of various parameters is shown in Fig. 5. The total lung volume (Fig. 5A), as well as the volume of the parenchyma (Fig. 5B), did not differ between experimental groups. The total volume of alveolar airspaces per lung (Fig. 5C) was significantly reduced by the AdTGF-β1 treatment whereas the PEEP-level during mechanical ventilation had no effect on this parameter. However, a significant difference in septal wall thickness was observed between the AdCl and AdTGF-β1 groups ventilated with a PEEP of 8 cmH₂O (Fig. 5D). The fraction of collapsed septa and the number of open alveoli showed significant differences between the treatment groups AdCl and AdTGF-β1 but, similar to the volume of alveolar airspaces per lung, this parameter was not affected by PEEP applied during the 4 h of ventilation. To conclude, the PEEP applied during prolonged ventilation did not affect quantifications of lung structure including the fraction of collapsed septa, septal wall thickness, or number of open alveoli.

### Electron microscopy

**Fig. 6:**
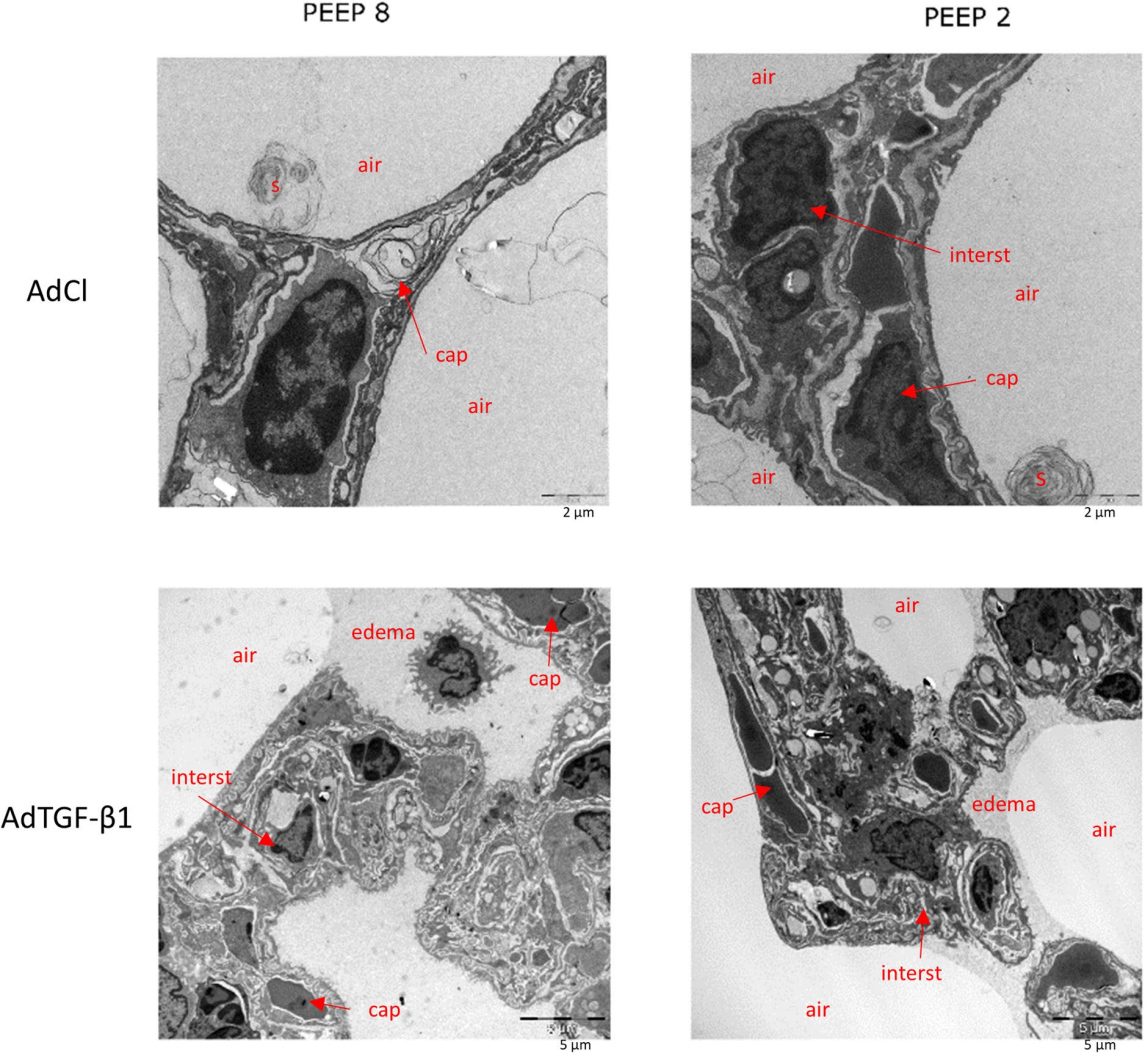
Ultrastructure captured by electron microscopy. The images show the intra-alveolar air (air), alveolar edema (edema) and secreted surfactant (s) as well as the septa including the capillaries (cap) and the interstitium (interst).

Figure 6 shows representative qualitative electron micrographs after 4 h of mechanical ventilation. Both control subgroups (top row) exhibited thin blood–gas barriers, presence of secreted surfactant within the alveolar airspace, and a basal lamina in a straight line. No qualitative differences were observed between the two PEEP levels. In contrast, the AdTGF-β1 subgroups (bottom row) displayed markedly thickened septa and an increased thickness of the blood–gas barrier due to a widening of the interstitium, e.g., because of interstitial edema and cell infiltration. The basal lamina appeared more irregular and less linear compared to that of the control mice. In addition, intra-alveolar edema could be observed adjacent to the septa. Like the control groups, no qualitative differences were detected between the two different PEEP levels.

### Biochemical analysis

**Fig. 7:**
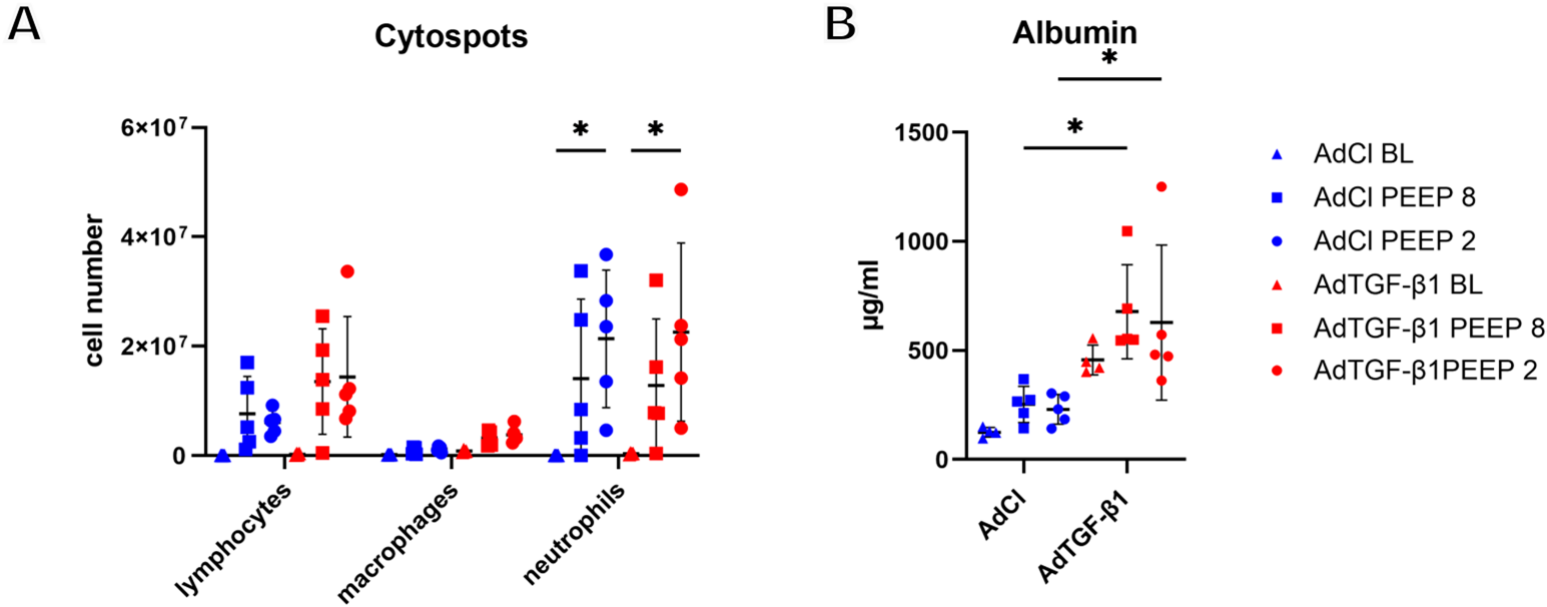
Biochemical analysis of the bronchoalveolar lavage. The cell types of cytospots (**A**) revealed an overall increase in the cell number of ventilated mice, with a significant rise in neutrophilic cells observed only in the PEEP2 subgroups compared with baseline (BL) mice. The albumin concentration (**B**) showed differences only between the treatment groups, although both parameters tended to increase in the ventilated mice independent from the PEEP values. For statistics, a one-way ANOVA was performed for albumin levels, and a two-way ANOVA for the cytospots. Statistically significant differences were indicated as follows: *p < 0.05, ** *p* < 0.01, *** *p* < 0.001.

Figure 7 presents this data in comparison with unventilated baseline (BL) mice. The analysis of BAL cell numbers revealed a significant increase in neutrophilic granulocytes in both AdTGF-β1 PEEP 2 and AdCl PEEP 2 subgroups compared to corresponding baseline lungs while no differences were detectable between PEEP 2 and PEEP 8 groups (Fig. 7A). In general, albumin concentrations were larger in AdTGF-β1 than in AdCl. Compared to corresponding unventilated lungs, the albumin concentrations were, moreover, elevated in ventilated mice in both AdCl and AdTGF-β1. However, an effect of the PEEP value during mechanical ventilation on the albumin concentration was not found.

### Transcriptomics

**Fig. 8:**
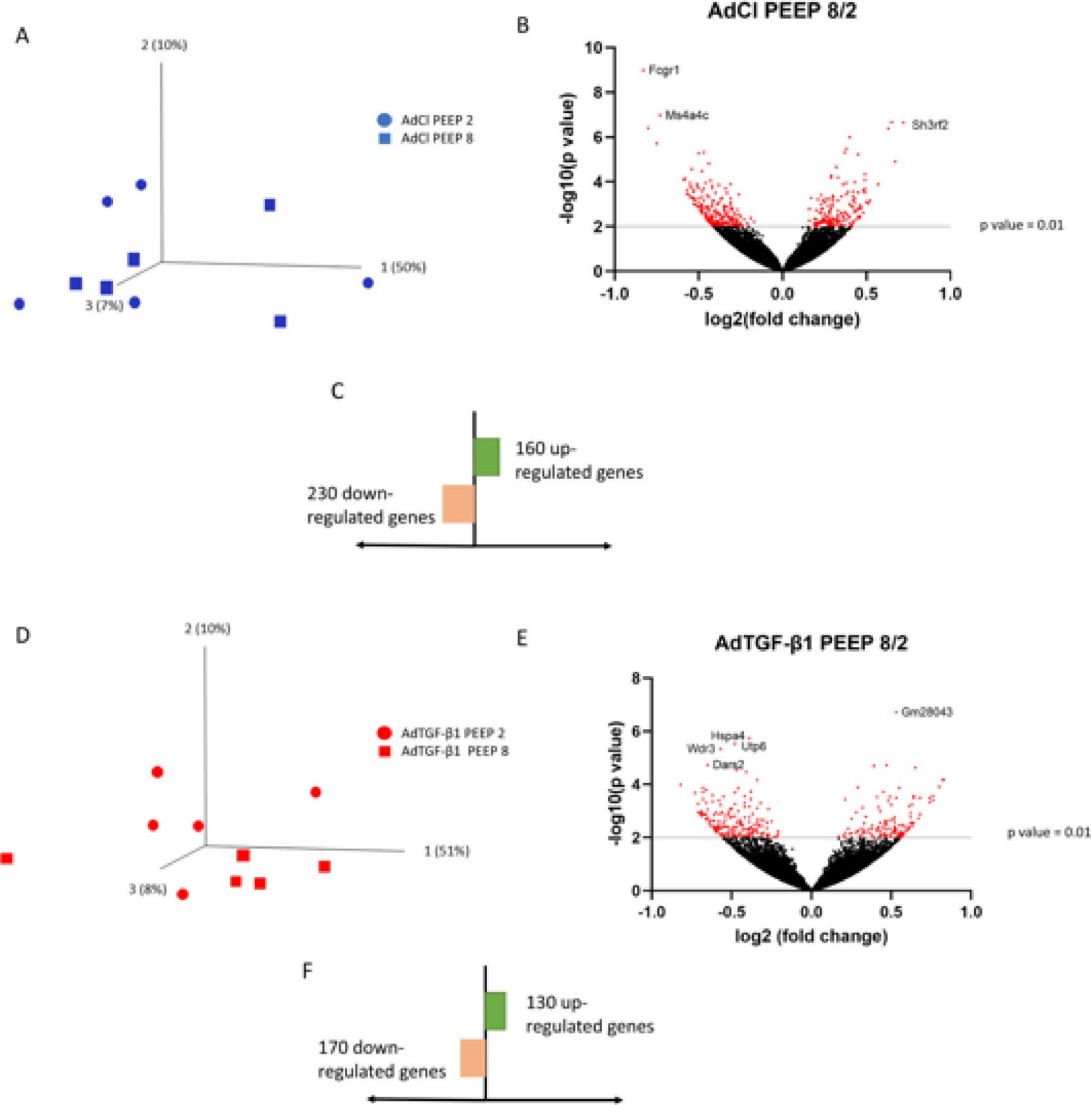
Transcriptomic analyses investigating the effects of PEEP within AdCl and AdTGF-β_1_ cmH_2_O groups. Principal component analysis (PCA) revealed no distinct clustering between the two PEEP groups, neither in the AdCl (**A**) nor in the AdTGF-β1 (**D**) animals. The volcano plots display significantly regulated genes (**B**,**C**,**E**,**F**), with 390 and 300 genes showing significant differential expression.

Panels A–C in Fig. 8 show the comparison between AdCl PEEP 8 and AdCl PEEP 2, while panels D–F illustrate the comparison between AdTGF-β1 PEEP 8 and PEEP 2. The principal component analysis (PCA) of both treatment groups did not reveal clear separations or distinct clustering in either the PEEP 2 cmH_2_O or the PEEP 8 cmH_2_O groups. The volcano plots highlight significantly regulated genes in red. In the AdCl group, approximately 230 genes were significantly downregulated and 160 were upregulated, whereas in the AdTGF-β1 group, approximately 130 genes were significantly upregulated and 170 were downregulated.

**Fig. 9:**
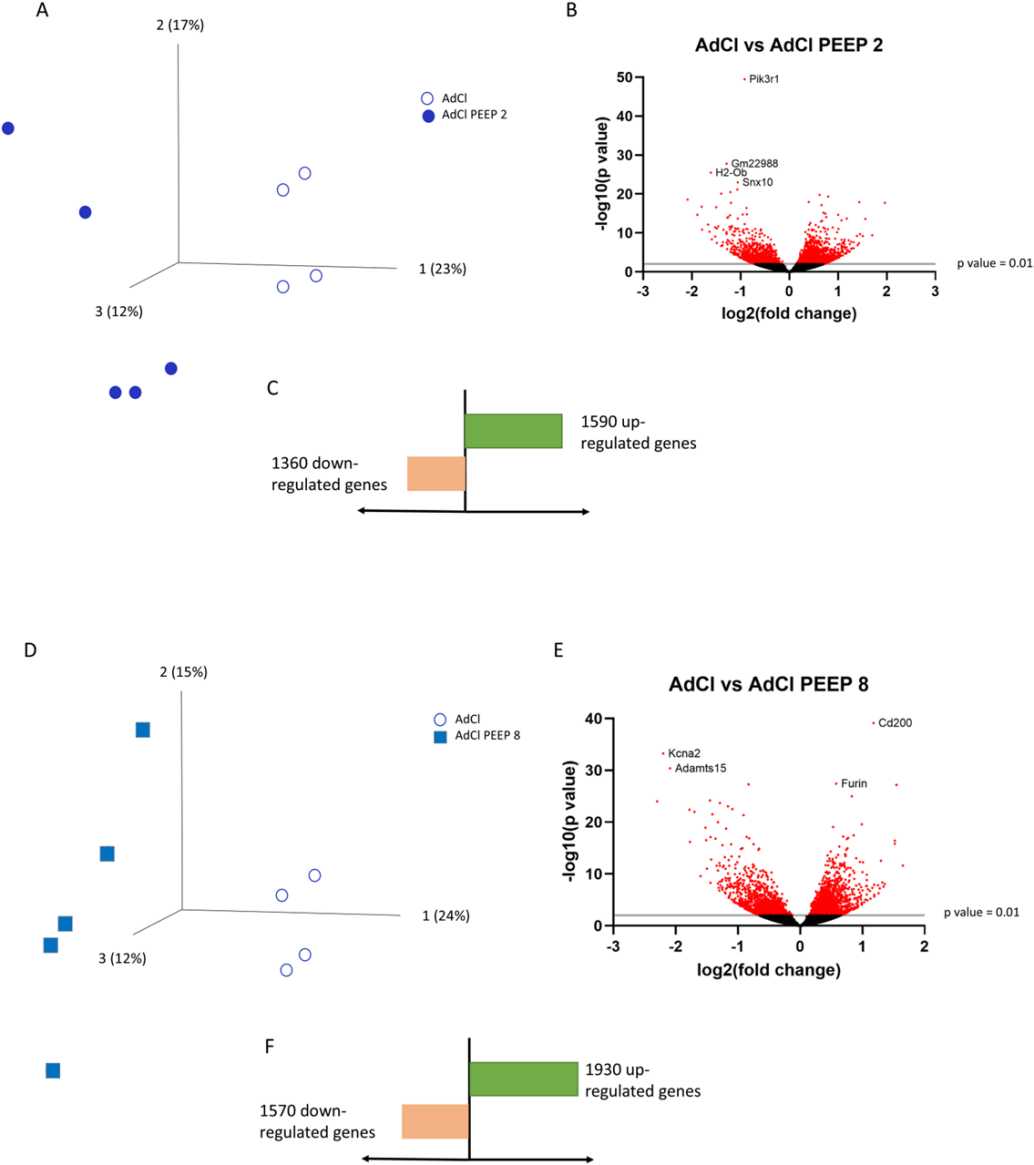
Transcriptomic analyses of non-ventilated AdCl compared with the ventilated groups. Principal component analysis (PCA) revealed two distinct clusters in each comparison (**A**,**D**). The volcano plots highlight significantly regulated genes in red (**B**,**E**) corresponding to the up- and downregulated genes in (**C**,**F**).

**Fig. 10:**
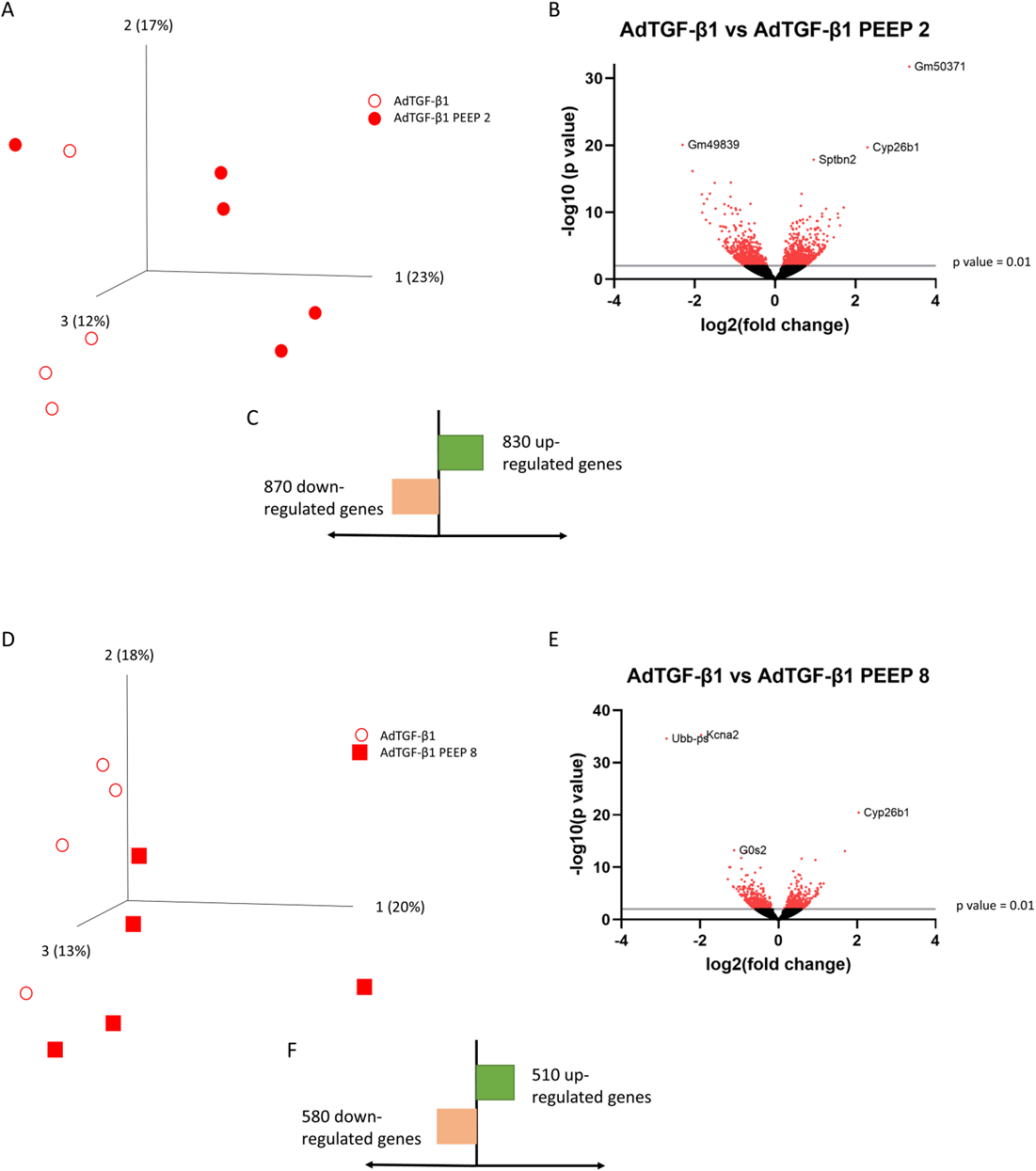
Transcriptomic analyses of non-ventilated AdTGF-β_1_ compared with the ventilated groups. Principal component analysis (PCA) revealed two distinct cluster in the PEEP 8 comparison (**A**,**D**). The volcano plots highlight significantly regulated genes in red (**B**,**E**) corresponding to the up- and downregulated genes in (**C**,**F**).

Only a few genes were differentially regulated in the comparison between different PEEP levels. However, both the AdCl and AdTGF-β1 groups, independent from PEEP-level, developed a phenotype such as a worsening of tissue elastance, BAL neutrophils, and BAL albumin. Hence, we compared each PEEP group with an unventilated baseline group from previous experiments^16^. In the control mice, comparisons revealed distinct clustering of ventilated and non-ventilated samples (Fig. 9). Numerous genes were significantly regulated, indicating that mechanical ventilation had a substantial impact on the transcriptome of the mice. A comparable trend was observed in the AdTGF-β1 mice, where the number of regulated genes was lower (Fig. 10). However, there were still more regulated than in the comparisons among the PEEP groups.

## Discussion

This study investigated the protective potential of mechanical ventilation of PEEP = 8 cmH_2_O in comparison to PEEP = 2 cmH_2_O in the TGF-β1 mouse model of lung injury. While a PEEP = 8 cmH_2_O could stabilize maximal airway opening pressures and tissue elastance during a period of 4 h of ventilation, a PEEP = 2 cmH_2_O resulted in a considerable increase in lung stiffness in AdTGF-β1 challenged lungs. However, these lung mechanics data were completely reversible by deep inflations administered with a periodicity of 30 min so that at the end of the ventilation protocol no PEEP effects were detected in terms of structural and lung mechanical data or markers of alveolocapillary leak and pulmonary inflammation. In other words, the pathology linked with low PEEP-ventilation induced transient lung mechanical abnormalities in AdTGF-β1 mice was reversible by deep inflations without any signs of persistent lung injury aggravation.

One week after intratracheal adenoviral-mediated gene-transfer of active TGF-β1 to the lungs, previous publications demonstrated that lung mechanical impairment was highly correlated with the occurrence of collapsed alveoli forming microatelectases^16,26,27^. The burden of microatelectases could be considerably reduced by recruitment maneuvers and return to an appropriate PEEP. Based on structure-function correlations, a PEEP of 8 cmH_2_O balanced the conflicting effects of increasing pressures: alveolar recruitment was achieved without an undue increase in static strain of acinar airspaces. Notably, both low and high PEEP levels were associated with increased tissue elastance linked to increased percentage of collapsed septa at low PEEP or dilatation of acinar airspaces at high PEEP^16^. In other words, PEEPs < 8 cmH_2_O increased microatelectases while PEEPs > 8 cmH_2_O were associated with increased static strain and lung stiffness^16^. Both of those conditions might trigger injury progression during mechanical ventilation based on the mechanisms of atelectrauma, volutrauma and stress concentration resulting in heterogenous ventilation^6,16,28^. For this reason, the conclusion of Roeder et al. 2024 was that a moderate PEEP = 8 cmH_2_O should prevent atelectrauma and stress concentration without an increased risk for volutrauma that would be associated with a too low and too high PEEP, respectively.

Accordingly, this current study investigated the effects of 4 h ventilation in healthy and pre-injured mice, ventilated either with the predicted protective PEEP level of 8 cmH₂O or the potentially harmful PEEP level of 2 cmH₂O. The lung mechanics measurements during the ventilation period in pre-injured AdTGF-β1 animals support the assumption that PEEP = 2 cmH_2_O represents the more harmful ventilation strategy. The driving pressure has been shown to be a predictor of poor outcome in clinical and pre-clinical studies^29,30^. Although driving pressures as defined in the clinical setting as the difference between end-inspiratory plateau pressure (usually measured during an inspiratory hold) and PEEP were not determined in the present study, we calculated ΔP as the difference between peak inspiratory pressure given in Fig. 3 and PEEP that could be considered as a dynamic approximation of driving pressure and depends on both resistive properties of the purely conducting airways and elastic properties of lung parenchyma. While ΔP was quite stable over time in AdCl groups and AdTGF-β1 PEEP 8, it showed a steeper increase and finally largest values in AdTGF-β1 PEEP 2 group, an observation that correlated with the behavior of tissue elastance, a marker reflecting properties of lung parenchyma. Unlike tissue elastance, the resistance Rn reflects the properties of the conducting airways and did not show a correlative time course so that the dynamic increases in ΔP was most likely a result of pathological changes in lung parenchyma and thus in support of the concept of our previous study that PEEP = 8 cmH_2_O is likely to be more protective than PEEP = 2 cmH_2_O in the AdTGF-β1 animal model.

A recent animal study, using endotoxin instillation to induce lung injury before mechanical ventilation with different PEEP values ranging from 1 to 8 cmH_2_O assigned the initial dynamic changes in driving pressures a central role to predict VILI. In that study a PEEP of 8 cmH_2_O was most protective for the pre-injured lung^29^. In the present study, dynamic changes in ΔP were most pronounced at the end of the ventilation period in AdTGF-β1, a finding that might indicate worsening of lung injury due to mechanical ventilation if duration would have been prolonged.

An increase in tissue elastance as well as maximal inspiratory pressure (Pmax) was observed over the course of the ventilation in the AdTGF-β1 PEEP2 group in this study. Every 30 min, however, ventilation was briefly paused for two deep inflations, which caused a substantial decrease in tissue elastance and Pmax, nearly returning these values to baseline levels. Following each maneuver, tissue elastance increased again, a finding that was most pronounced in the AdTGF-β1 PEEP 2 group. That considerable increase in lung stiffness was most likely a consequence of progressive alveolar derecruitment with time in AdTGF-β1. The initial stage of TGF-β1 gene transfer exhibits high surface tension linked with lack of surfactant proteins (SP-) B and C within the alveolar space increasing the risk of alveolar collapse at low lung volumes^27^. Tissue elastance has been shown to correlate inversely with the number of open alveoli in animal models of lung injury so that progressive loss of alveoli with time appears to be a plausible explanation for the increase in H^6,26,31,32^. The observation that lung mechanical impairments are reversible by deep inflations suggests that, in this case, alveolar collapse is also reversible, at least temporally. This invertibility is supported by the stereologically-analyzed morphology. The fixation of the lung was performed following a deep inflation so that the stereological data describes the parenchyma after recruitment. Here, Pmax and tissue elastance were effectively reduced in all study groups and most strongly in the AdTGF-β1 PEEP 2 subgroup. While clear effects of the TGF-β1 gene transfer on stereological data could be identified, the applied PEEP did not have an impact on the number of open alveoli or the fraction of derecruited septa. However, at low PEEP in the pre-injured AdTGF-β1 group it seems that the effective recruitment of alveoli was only temporally so Pmax and tissue elastance increased considerably as a function of time till the next deep inflation. Thus, progressive alveolar instability appeared to be most distinctive in AdTGF-β1 mice ventilated with PEEP = 2 cmH_2_O, an observation that is likely explained by high surface tension due to surfactant dysfunction.

Collapsed alveoli forming (micro)atelectasis function as stress concentrators that may increase strain in adjacent open alveoli^37^. Increased strain, moreover, has been shown to cause surfactant dysfunction e.g., by fostering conversion of biophysically active large aggregates to inactive small aggregates^38,39^. In conditions of surfactant dysfunction, alveoli have also been shown to be subject to repetitive recruitment and expansion on inspiration and subsequent derecruitment on expiration (= atelectrauma). These large fluctuations in alveolar volume have been suggested to result in ruptures of the surfactant monolayer at the air-liquid interface^40,41^. After rupture of the surfactant films, albumin, which is elevated in the alveolar space after AdTGF-β1 challenge, gets the opportunity to compete against phospholipids of the alveolar surfactant for access to the air-liquid interface so that surface tension further increases and propagates additional alveolar instability^42,43^. Taken together, collapsed alveoli might be subject to atelectrauma or act as stress concentrator to induce regional volutrauma and both mechanisms have the potential to inactivate surfactant. Hence, it is possible that PEEP = 2 vs. 8 cmH_2_O ventilation might result in a local volutrauma next to atelectatic regions and/or atelectrauma, aggravating surfactant inactivation and additional alveolar instability linked with increase in tissue elastance as well as peak inspiratory pressure. Of note, unlike tissue elastance, dynamic compliance measured during invasive mechanical ventilation did not clearly distinguish AdTGF-β1 PEEP 8 from AdTGF-β1 PEEP 2.

The lung mechanics and stereology show that low PEEP ventilation of AdTGF-β1 pretreated mice in comparison to ventilation with higher PEEP does not cause more permanent damage to the lung while the pathology underlying transient deterioration of lung mechanics seems to be reversible by deep inflations. The lack of permanent injury may be explained by a comparatively short ventilation period of four hours that might not suffice to cause detectable differences in injury severity between AdTGF-β1 groups ventilated with low and higher PEEP. This reasoning is supported by the observation in AdTGF-β1 mice that the course of peak inspiratory pressure got much steeper, the gap in ΔP between the different ventilation protocols larger and thus PEEP = 2 cmH_2_O ventilation potentially more injurious towards the end of the protocol. Hence, a prolongation of mechanical ventilation could have produced different degrees of permanent lung injury in the study groups. In a recent publication using a 2-hit model to compare the injurious potential of different PEEP-levels, the injury markers including stereological data were very stable for four hours while peak inspiratory pressures and tissue elastance showed a similar dynamic time course as described in the present study. However, between 4 and 6 h of mechanical ventilation an accelerated degeneration of structural and functional parameters including vascular leakage could be observed^6^.

Additionally, the deep inflations might promote, surfactant release by alveolar epithelial type 2 cells via stretch-induced mechanisms^44^ as well as by restoring the surfactant monolayer at the air-liquid interface by the adsorption of fresh phospholipids from the surfactant reservoir located in the alveolar hypophase^35^. Accordingly, the deep inflations may exert an influence on elastance through secretion and maintenance of surfactant^36,45^. In the PEEP 2 group, deep inflations could cause recruitment of collapsed alveoli along with triggering surfactant release, rendering the effects of newly secreted surfactant more pronounced than in the PEEP 8 group. Electron microscopy shows that secreted surfactant is clearly visible in the healthy group. In the AdTGF-β1 group, thickened septa, a widened blood–gas barrier and a protein-rich intra-alveolar edema is visible, reproducing observations from previous studies^26,46^. This intra-alveolar, albumin-containing edema is likely to inactivate surfactant so that appropriate maintenance of surfactant after AdTGF-β1 challenge might be dependent on deep inflations.

Biochemical analysis of the BAL fluid found increased albumin concentrations, a marker of alveolocapillary leak, in AdTGF-β1 mice that follows previous findings^16^. However, no difference between PEEP = 8 and 2 cmH_2_O was observed, indicating that the applied PEEP did not influence the degree of vascular leakage during four hours of ventilation. Volutrauma and/or atelectrauma cause significant tissue stretch, weakens cell–cell contacts, and damages the blood–gas barrier^47^, potentially allowing cytokines or other substances to enter the circulation^48^. Pulmonary cells such as the alveolar epithelial type 2 cells secrete interleukins as a response to increased stretch^49^ or even rupture of alveolar epithelial cells‘ plasma membranes that would then lead to an influx of granulocytes^50^. On the other hand, type I cells require stretch to maintain normal function, as they are mechanosensitive and may otherwise be reprogrammed into type 2 cells in the absence of sufficient mechanical stimulation^51^. Based on cytological data, ventilation appears to have an impact on cytokine-mediated influx of neutrophilic cells into the alveolar space. Both treatment groups exhibited a significant rise in neutrophils that was PEEP-independent. However, transcriptome analyses comparing non-ventilated and ventilated animals reveal no significantly regulated genes within neutrophil immune response pathways. The comparisons did reveal other differentially regulated genes including oxidative stress (HMOX1), adhesion molecules (ICAM1), and apoptosis (BCL2) pathways when comparing the non-ventilated AdTGF-β1 and the AdTGF-β1 PEEP 2 groups.

Consistent with the other findings, the principal component analyses (PCA) of transcriptome data comparing the different PEEP levels showed no distinct clustering associated with PEEP levels. Only a small subset of genes was significantly regulated—390 in control animals and 300 in AdTGF-β1 mice—indicating that the effects of different PEEP levels on gene expression profiles are minimal. In the present study, the AdTGF-β1 challenge as such has a much stronger effect on structural data and lung mechanics and therefore also dominates the transcriptome profile in lung parenchyma^16^ whereas the effects related to the different PEEP levels are comparably weak. Other studies in which a clearly non-revertible functional or inflammatory phenotype was induced by mechanical ventilation have identified differences in gene expression profiles in dependence on the ventilation parameters such as upregulation of genes related to inflammation^52^. However, the PEEP levels and tidal volumes employed are not comparable to the present study, making it difficult to draw meaningful parallels. Moreover, in Dolinay et al.^52^, initially healthy lungs were ventilated, and deep inflations were administered only during the first out of four hours. Beyond their role in preventing atelectasis, deep inflations additionally help to reduce inflammation^53^.

The beneficial effect of deep inflations in the animal model used in the present study is likely due to the high recruitability of alveolar airspaces, a feature that strongly depends on the injury model and the degree of injury^19,20,54^. The mouse model of SP-B deficiency also generates high surface tension-induced lung injury^55,56^ and a lack of deep inflations during ventilation resulted in increased volume of protein-rich alveolar fluid and accumulation of intracellular surfactant within alveolar epithelial type 2 cells^57^. With progression of lung injury and the occurrence of fibrin within the alveolar space the potential to open collapsed alveoli is reduced so that the protective effect of deep inflations is limited^54^. In the present study, however, the electron microscopic investigation did not provide any evidence of fibrin deposition within the lung, suggesting that the potential to recruit alveolar airspaces remained stable till the end of the ventilation period in all study groups.

Clinical application of the findings observed in animal models showing the benefits of deep inflations has remained elusive given, among other factors, that recruitability of distal airspaces is highly patient-dependent. According to imaging studies in ARDS patients, those with high can be distinguished from those with a low potential for recruitability^58^. Nevertheless, a study could show that due to short cyclic recruitment maneuvers, the ventilation heterogeneity was decreased^59^. Another clinical study preventively applied sighs in patients at high risk for the development of an ARDS during mechanical ventilation at the intensive care unit and found a trend for an improvement in clinical outcome^60^. Overall, additional studies in the intensive care setting are essential to investigate the efficacy of deep inflations in particular patient groups. At present, the data are too inconsistent to support the routine use of deep inflations^61^.

This study has several limitations. First, only female mice were used in the experiments to reduce the variability in the stereological data, that are usually referred to the total lung volume, which is highly sex-depend, to avoid the reference trap. While a comparison with male mice would have been valuable to understand whether male mice react differently from female mice, including both sexes would have required approximately twice the number of animals. Second, transcriptome analyses of the non-ventilated animals were conducted prior to those of the ventilated group. Although the data were generated using the same conditions including the RNA-sequencing depths, the temporal separation in data acquisition may have introduced inconsistencies, potentially affecting the precision of cross-group comparisons. Third, the lungs were fixed by vascular perfusion after a deep inflation at an airway opening pressure of 5 cmH_2_O on expiration. To establish structure-functions relationships and to understand the structural basis of the worsening of lung mechanical data in AdTGFβ1-PEEP 2 fixation of the lungs without prior deep inflation would have been important. By fixing the lungs after a deep inflation we were only able to identify those pathological alterations that were not reversible by a deep inflation.

In conclusion, in AdTGF-β1 animal model the PEEP = 2 cmH_2_O ventilation strategy was associated with highly dynamic changes over time such as the increases in tissue elastance, peak inspiratory pressure and thus ΔP. These dynamic changes were markedly mitigated by the application of a PEEP = 8 cmH_2_O, indicating that this PEEP level represents a gentler ventilation strategy. The morphological and biochemical analyses revealed no relevant difference between the two ventilation protocols. These findings indicate that, within our 4-hour ventilation protocol with deep inflations performed every 30 min, a PEEP level of 2 cmH_2_O with a high burden of micoatelectases did not produce more permanent and discernible effect on lung tissue compared to a PEEP level of 8 cmH2O with a much lower burden of microatelectases.

## Supplementary Information

Below is the link to the electronic supplementary material.

Supplementary Material 1

Supplementary Material 2

Supplementary Material 3

Supplementary Material 4

Supplementary Material 5

Supplementary Material 6

## Data Availability

Galaxy files of the transcriptom analyses have been added to the online supplement of this manuscript. The source data of the bulkRNA-sequencing are now available via the Zenodo repository (doi: 10.5281/zenodo.21037035). The other source data of this study will be made available by the corresponding author upon reasonable request.
